# *Achromobacter xylosoxidans* Relapsing Peritonitis and* Streptococcus suis* Peritonitis in Peritoneal Dialysis Patients: A Report of Two Cases

**DOI:** 10.1155/2018/9454520

**Published:** 2018-07-30

**Authors:** Rafał Donderski, Magdalena Grajewska, Agnieszka Mikucka, Beata Sulikowska, Eugenia Gospodarek-Komkowska, Jacek Manitius

**Affiliations:** ^1^Department of Nephrology, Hypertension and Internal Medicine, Ludwik Rydygier Collegium Medicum in Bydgoszcz, Nicolaus Copernicus University in Toruń, Poland; ^2^Department of Microbiology, Ludwik Rydygier Collegium Medicum in Bydgoszcz, Nicolaus Copernicus University in Toruń, Poland

## Abstract

Peritonitis is considered to be the most common complication of peritoneal dialysis (PD). It is usually caused by Gram positive* Staphylococcus epidermidis. Achromobacter xylosoxidans (A. xylosoxidans) and Streptococcus suis (S. suis)* are rare pathogens, but there is emerging evidence that they may be also responsible for PD related peritonitis. We described 2 cases of rare peritonitis treated in our center. In our opinion this is the first described case of PD related peritonitis caused by* Streptococcus suis.*

## 1. Introduction

Peritoneal dialysis (PD) is a worldwide used modality of renal replacement therapy (RRT). Disadvantages of this kind of treatment are infectious complications such as peritonitis, exit site infection (ESI), or tunnel infection. Peritonitis contributes to increased hospitalization rate, technique failure, and increased mortality in PD patients. Nowadays, according to novel connectivity systems and impact on educational training of patients the annual incidence of a new peritonitis episode seems to be decreased [1].* Achromobacter xylosoxidans *(formerly* Alcaligenes xylosoxidans*) infections are extremely rare in PD patients. This bacterium was first described in 1971 by Yabuuchi and Ohyama in patients with chronic otitis media [2]. It is mostly detected in immunocompromised patients such as diabetic patients, chronic kidney disease (CKD) patients, or individuals undergoing chemotherapy.* S. suis* is a pig pathogen and it is rarely identified in humans. There is emerging evidence of* S. suis* infections such as meningitis, peritonitis, and septic shock in especially immunodeficient patients [3]. In our center we reported a rare case of* A. xylosoxidans* relapsing peritonitis and a case of* S. suis* peritonitis.

## 2. Patient 1

27-year-old woman who had been on PD because of chronic glomerulonephritis confirmed by renal biopsy (histopathological evaluation revealed focal segmental glomerulosclerosis (FSGS)) and end-stage kidney disease (ESKD) for 2 years was admitted to our center with clinical symptoms of peritonitis. She was complaining of diffuse abdominal pain, fever, and cloudy dialysate. There were no signs of exit site infection (ESI). She was on automated peritoneal dialysis (APD) using Home Choice Pro device delivered by Baxter (USA). Her dialysis regimen was 12.0 liters of 1.36% Dianeal fluid. Her ultrafiltration was approximately 1000ml. Residual diuresis was approximately 1.0 liter daily. She had not had any PD-associated infections in the past. Moreover, she was suffering from chronic hepatitis C, hypertension, reflux esophagitis, and chronic gastritis. She had renal anemia treated with darbepoetin alfa s.c. and chronic kidney disease—mineral and bone disorder (CKD-MBD) were treated according to latest updated KDIGO Guidelines 2017 (calcium carbonicum in a dose of 3 x 3,0g daily, cinacalcet in a dose of 30mg daily, paricalcitol 2,0mcg 3 times a week). In 1990 she had episode of hemolytic-uremic syndrome (HUS) and acute kidney injury (AKI stage 3) with need for dialysis (PD was used for a month that time). Her vital signs on admission were as follows: she presented generalized abdominal tenderness, her blood pressure was 130/90mmHg, her temperature was 38°C, heart rate was 100 beats per minute, and respiration rate was 20 per minute. Laboratory test included white blood count (WBC) of 15.000/mm^3^, hemoglobin (Hgb) of 12,1g/dl, platelets count of 254.000/mm^3^, C-reactive protein (CRP) of 84,5mg/L, blood urea nitrogen (BUN) of 65mg/dl, serum creatinine of 4,15mg/dl, serum albumin of 3,8g/dl, total protein of 6,58g/dl, total cholesterol of 220mg/dl, dialysate leukocyte count (DLC) of 7217/mm^3^ (neutrophils/lymphocytes=86% vs 11%), PTH (parathormone) of 984pg/ml, serum calcium of 2,10mmo/l, and phosphorus of 1,86mmo/l. Blood culture and urine culture were both negative. We decided to start initial empiric antibiotics treatment with ceftazidime 1,0g daily and cefazolin 1,0g daily infused intraperitoneally (ip) into Extraneal 2,0-liter bag. Dwell time was 8 hours. Positive culture of* A. xylosoxidans* colonies was isolated from peritoneal fluid (using BD Bactec™ FX Becton Dickinson System, USA). Isolation of bacteria was performed using routine method at the clinical microbiology laboratory. The identification of isolated strain was further confirmed by applying mass spectrometric method (Matrix-Assisted Laser Desorption/Ionization-Time-Of-Flight, MALDI-TOF) using MALDI Biotyper (Bruker, Germany). Antimicrobial susceptibility testing of examined* A. xylosoxidans* strain was performed by the agar dilution method and carried out according to the European Committee on Antimicrobial Susceptibility testing recommendation (version 7.0). According to the antibiogram, the pathogen was sensitive to piperacillin/tazobactam, ceftazidime, and imipenem and was resistant to ciprofloxacin and cefepime. The treatment regimen was changed according to antibiogram results into imipenem+cilastatin intravenously (iv) in a dose of 2 x 0,25g daily. She was given the antibiotic for 2 weeks. Laboratory investigation and antibiotics regimen in the first episode of* A. xylosoxidans* peritonitis are given in Table 1.

We observed improvement in her clinical condition. There was no abdominal pain. Dialysate leukocyte count gradually decreased and control dialysate culture was negative. 3 weeks after the treatment, she was discharged from hospital. 7 days later she was admitted again because of another episode of peritonitis. Dialysate culture was positive and* A. xylosoxidans* colonies were identified again. Pathogen was sensitive to imipenem and ceftazidime and we started these antibiotics in a dose of 2 x 0,25g iv and 1 x 1,0 g intraperitoneally (ip), respectively. This time we decided to perform computed tomography (CT). There were no signs of gut perforation or abdominal abscess. After antibiotics implementation her symptoms disappeared quickly. We continued the treatment in the hospital for another 2 weeks. 2 weeks after being discharged from the hospital, there was another third episode of peritonitis with the same pathogen,* A. xylosoxidans* isolated from peritoneal fluid. In the case of relapsing* A. xylosoxidans* peritonitis, we decided to remove peritoneal catheter and transfer her to hemodialysis. After permanent catheter insertion into right carotid vein and control chest X-ray, she started temporary hemodialysis treatment. She was on 3 hemodialysis sessions per week. The tip of removed peritoneal catheter was sent for microbiological evaluation. Colonization of* A. xylosoxidans* was stated. After 2 months on hemodialysis, we decided to continue PD in her case and she was admitted to Department of Surgery for peritoneal catheter insertion. Then, 3 weeks later she successfully started PD treatment again.

## 3. Patient 2

The patient had been a 54-year-old male with CKD stage 5 secondary to multiple myeloma (MM). He was on PD since November 2015. After 2 months on CAPD he started APD using Fresenius Sleep-Safe Cycler. His dialysis regimen was 12,0 liters of 1,5% glucose solution. He had well preserved residual renal function (RRF) with residual diuresis approximately 1,5 liters daily. His ultrafiltration rate ranges from 600 to 800ml daily. Moreover, his medical history included diabetes type 2, hypertension, psoriasis, hernia esophagi, peptic ulcer, and spinal column rupture (compression rupture Th5-Th8) related to MM. On admission to the hospital he presented mild abdominal pain and turbid dialysate. Physical examination revealed the following: his temperature was 37,5°C; his pulse rate was 78 beats/min; blood pressure was 120/70mmHg; respiration rate was 18 per minute; his abdomen was tender to palpation with positive Blumberg sign. Laboratory tests were as follows: WBC 8,280/mm^3^, Hgb 7,8g/dl; platelets count 307.000; CRP 71,38mg/L; BUN 66,2mg/dl; serum creatinine 7,02mg/dl; serum albumin 3,1g/dl; total protein 5,3g/dl; total cholesterol 228mg/dl; dialysate leukocyte count 530/mm^3^ (neutrophils/lymphocytes 78% vs 10%), serum calcium 1,77mmo/l; phosphorus 2,1mmo/l; sodium 139,1mmo/l; potassium 3,9mmo/l. Samples of peritoneal fluid, blood, and urine were inoculated. Growth of* S. suis* from peritoneal dialysis fluid was confirmed by our clinical microbiology laboratory. Blood culture and urine culture were negative. Current methods for serotyping a strain of* S. suis* are serology, PCR using specific primers (for cps genes) or whole-genome sequencing, and analysis of the cps genes, which were not available in the laboratory. The strain was identified using MALDI-TOF MS technique on MALDI Biotyper apparatus (Bruker) as described above.* S. suis* colonies were sensitive to penicillin G, ampicillin, cefotaxime, ceftriaxone, imipenem, and clindamycin, as described above. After 2 days of empiric intraperitoneal antibiotics administration (cefazoline+ceftazidime ip), we changed the treatment according to antibiogram results into ceftriaxone iv. We continued ceftriaxone iv 2,0g per day for 2 weeks. His clinical condition improved. There was no abdominal pain; peritoneal fluid was transparent. Control peritoneal fluid culture was negative. After 2 weeks of hospital treatment the patient was discharged. In his case we did not observe relapse of peritonitis.

## 4. Discussion

### 4.1. *A. xylosoxidans* PD Related Peritonitis

*A. xylosoxidans* is Gram negative, nonfermenting, aerobic, oxidase- and catalase-positive bacterium. Natural environments of* A. xylosoxidans* are soil and water.* A. xylosoxidans* is also a part of normal human flora. It can be encountered in dairy products. The clinical presentation of* A. xylosoxidans* infections is pneumonia, meningitis, urinary tract infection, endocarditis, bacteremia, and catheter related infections especially in immunocompromised patients. This bacterium is also detected in skin and gastrointestinal tract as normal human flora [4].* A. xylosoxidans* infection in PD patients is very rare. According to literature data, 11 cases of* A. xylosoxidans* peritonitis have been described so far. These cases were reported mainly in diabetic kidney disease patients and primary glomerulonephritis (IgA nephropathy). Moreover, 2 cases of ESI caused by* A. xylosoxidans* were described. Jun-Li et al. reported first case of* A. xylosoxidans* related tunnel infection in a patient receiving PD. PD related peritonitis was also diagnosed in this patient.* A. xylosoxidans* related tunnel infection was confirmed in CT. The Tenckhoff catheter was removed and new one was inserted at the opposite site [5]. The risk factor for* A. xylosoxidans* infection is CKD per se according to its immunocompromised nature. Aqueous environment and glucose containing PD solution facilitate* A. xylosoxidans* infections.* A. xylosoxidans* colonies can form biofilm on PD catheters and therefore removal of infected catheters is the best method of treatment [5, 6]. In our patient, because of* A. xylosoxidans* relapsing peritonitis removal of PD catheter was performed. After she was transferred to hemodialysis, we continued antibiotic administration for the following 2 weeks. It is worth mentioning that* A. xylosoxidans *infections may occur in long-term hemodialysis patients especially in these having permanent catheters. Moreover, risk factors for* A. xylosoxidans* infections are poor socioeconomic status, contact with animals, poor personal hygiene, and contamination of catheters. In HD patients source of* A. xylosoxidans* infection comprises catheters presence, use of the heparin multidose vials, the dialysate itself, and hands and clothes of healthcare staff [7].

### 4.2. *S. suis* PD Related Peritonitis

Another peritonitis reported in our PD center was peritonitis caused by* Streptococcus suis*. This bacterium is common in pigs, piglets, and farm animals. It is also detected in ruminants, dogs, cats, deer, wild boars, and horses. Almost all pigs and farms worldwide are* S. suis* carriers.* S. suis* infection is common zoonosis especially in Southeast Asia. In Hong Kong* S. suis* meningitis is the most frequent bacterial meningitis in adults. There is an increasing evidence of* S. suis *infections in North America and Western countries. In France and United Kingdom* S. suis* infection is recognized as an industrial disease.* S. suis* may cause serious, even life threating diseases in humans. Meningitis and septicemia related to* S. suis* infection were first described in 1968 in Denmark [8]. Clinical presentation of* S. suis* infection is as follows: meningitis, pneumonia, arthritis, endophthalmitis, endocarditis, spontaneous bacterial peritonitis, skin lesions, bacteremia, and septic shock [3, 9]. Partial or complete deafness is an important complication of* S. suis* meningitis. The risk of this zoonosis is related to exposure to pigs (people at risk are farmers, veterinarians, and truck drivers who have direct contact with infected pigs) and pork-derived products (people handling fresh pork processing, people handling carcasses, people such as slaughterhouse workers, butchers, and people handling raw meat at home for cooking).* S. suis* infection is almost absent in children and is highly reported in men, with very high male to female ratio. It is generally considered as an occupational disease [8, 10, 11]. The main route of entry of* S. suis* is through contact of cutaneous lesions (on hands, arms) with contaminated animals, carcasses, or meat or oral route especially in Asian countries where infection has been reported after consumption of undercooked contaminated pork products. As wild boars are also carriers of* S. suis*, the cases of hunters that were infected while handling the carcasses were reported especially in France. In area of Barcelona in Spain, urban wild boars were considered to be a novel public health risk factor for nonhunters [12]. The real infection rate of* S. suis* remains unknown. Human microbiology diagnostic laboratories in Americas and European countries are not aware of this zoonosis and commonly misidentified* S. suis* as enterococci,* S. pneumoniae, S. bovis, S. viridans* group streptococci (*Streptococcus anginosus *and* Streptococcus vestibularis), *or* Listeria. *According to the available literature, serotype 2 ST1 is predominant in Europe. It does not present cross reactivity with other serotypes, in contrast to serotype 14, for example, [13]. The tested strain presented the closest similarity to the reference strain DSM 9682T, belonging to serotype 2, which was originally derived from the pig. Since the MS identifies microorganisms mainly on the basis of ribosomal proteins, in the epidemiological investigations of* S. suis* infections preferably serotyping and molecular methods should be used. MALDI-TOF MS presents 100% sensitivity and specificity for identifying but not serotyping of the most of the* Streptococcus* spp. [14] and enables discrimination between* S. suis* and* S. porcinus* and* S. dysgalactiae *subsp*. equisimilis*. In summary MALDI-TOF MS represents a rapid, accurate, and cost-saving method for routine identification of* S. suis* isolates from both human and animal origins [15].

Peritonitis caused by* S. suis* was previously described in literature but we did not find a description of* S. suis* peritonitis in PD patients. Spontaneous* S. suis* peritonitis was described in patient with alcoholic liver damage [16]. Another case of* S. suis* peritonitis was described in 45-year-old Thai male with acute kidney injury (AKI) stage 3 related to trauma and rhabdomyolysis [17]. Alcoholism, diabetes, malignancy, and contact with infected animals (especially pigs) are considered to be predisposing factors for* S. suis* peritonitis. Our patient was mean age immunocompromised male with MM and CKD. He declared that he did not have any contacts with animals especially pigs or wild animals. He was not occupationally handling meat; he had retired a few years ago. No one from his relatives occupationally was handling meat products. In the past he worked as a sales representative of chemical company. He denied consuming fresh, undercooked pork meat. He denied any serious cutaneous lesions as a route of infection. He was not a hunter. The course of infection in his case was mild and there was a good response for the treatment and no relapse. We suspect that there was a contact with infected animal or pork product used in cooking that patient did not realized, while he was spending his weekends in the rural area. The possible route of infection in his case was improper hand washing before connecting to the cycler which is quite common in PD patients. In our opinion this is the first description of* S. suis *peritonitis in PD patient.

## 5. Conclusions

Peritonitis caused by rare pathogens such as* A. xylosoxidans* and* S. suis* should always be taken into consideration in PD patients, especially in immunocompromised individuals with diabetic kidney disease, malignancies, or other serious underlying diseases. Specific environmental factors or some patients' habits may facilitate infections caused by described pathogens.

## Figures and Tables

**Table 1:** Laboratory investigation and antibiotics treatment in the first episode of *A. xylosoxidans* peritonitis in patient 1.

Laboratory tests	Antibiotics prescription
WBC – 15.000/mm^3^ (N: 4-11x10^3^/mm^3^)	(1) Ceftazidime 1,0g+cefazoline 1,0 IP*∗*
CRP -84,5mg/L (N<5,0)	(2) Imipenem 0,5g IV*∗*
DLC- 7217/mm^3^ (N<100)	

N: normal value; WBC: white blood count; CRP: C-reactive protein; DLC: dialysate leukocyte count; IP: intraperitoneally; IV: intravenously. *∗* means once a day.

## Data Availability

All information was obtained from available medical documentation.
